# Current Water Management of Small Lotic Waterbodies in the Context of Nature Conservation in Germany

**DOI:** 10.1007/s00267-023-01904-y

**Published:** 2023-11-04

**Authors:** Isabelle Idilbi, Axel Ssymank, Andreas Martens

**Affiliations:** 1 Institute for Biology, University of Education Karlsruhe, Karlsruhe, Germany; 2 Natura 2000/Habitats Directive, Federal Agency for Nature Conservation, Bonn, Germany

**Keywords:** Habitats Directive, Biodiversity, Agriculture, Water management, Knowledge exchange, *Coenagrion mercuriale*

## Abstract

Small lotic waterbodies are abundant and species rich habitats, offering refuges and microhabitats to protected species of the European Union Habitats Directive. Highly impacted by water management actions, it is essential to reveal the current status and challenges of water management. The present study aims to identify relevant issues by conducting a survey concerning water management authorities. Authorities were selected according to their involvement in the management of small lotic waterbodies within the actual range of a threatened species, *Coenagrion mercuriale* (Odonata), which is highly dependent on water management actions and protected by the Habitats Directive. The survey involved three sets of questionnaires, (1) socio-demographic (personal) questions (2) specific questions about water management and (3) questions on the biological background. Out of 181 selected authorities, 75 participated in the survey. The results showed that though nature conservation interests are partially considered, they represented a minor factor in water management decision-making. In addition, knowledge exchange is insufficient between involved stakeholders from policy, management practice and science, which was especially reflected in the case of equipment use and accruing material. The reconciliation of both, water management and nature conservation interests, can contribute to enhance the conservation status of key protected species of small lotic waterbodies under the Habitats Directive.

## Introduction

Small lotic waterbodies, (semi-)artificial and natural, contribute to biodiversity and ecosystem services (Biggs et al. 2017). They provide microhabitats and exhibit a variety of species (Armitage et al. 2003; Verdonschot et al. 2011), including uncommon to very rare taxa (Verdonschot 2012). Especially for species which lost their primary habitat, small lotic waterbodies become a refuge as a secondary one. The damselfly *Coenagrion mercuriale* (Charpentier 1840) is an example for those species, protected and covered by the Habitats Directive Annex II Council Directive 92/43/EEC (Council of the European Communities 1992 [Habitats Directive]; Sternberg and Buchwald 1999), which represents the Natura 2000 network together with the Birds Directive. The primary habitat of the threatened *C. mercuriale* are drains of calcareous fens which occurrence declined due to the beginning of fenland exploitation around the 17th century and finally led to massive destruction of fens in the 20th century (Succow and Jeschke 2022). After all, *C. mercuriale* colonized a different, but equivalent habitat type: sunny, little to moderate flowing small waterbodies with winter-green submerged water-vegetation (Wildermuth and Martens 2019), which themselves also represent a protected habitat type 3260 “Water courses of plain to montane levels with the Ranunculion fluitantis and Callitricho-Batrachion vegetation” of Annex I Habitats Directive.

The Habitats Directive was implemented in 1992 to reach a good nature conservation status and to maintain biodiversity, involving management plans as well for Natura 2000 sites. However, the state of nature report 2013–2018 identified that management actions exist for 60% of the habitat sites only, yet only a few are implemented since personal and financial resources are missing (European Environment Agency 2020). In this study, the habitat type 3260 and a variety of species (e.g. *Natrix tesselata, Lampetra planeri, Misgurnus fossilis, C. mercuriale, Coeagrion ornatum, Unio crassus*) are of interest according to the Habitats Directive (Ssymank et al. 2021). A favorable habitat conservation status includes per definition in Art. 1e of the Habitats Directive also a good status of its “typical species” (Art. 1i). However, animal species are still largely neglected in practical nature conservation and management of habitats. Many of these typical species have certain habitat requirements in common with the target species *C. mercuriale*, like slow currents and macrophytes. In addition, small lotic waterbodies which are part of a catchment area of >10 km² are covered by the European Water Framework Directive [WFD], which aims to reach a good ecological potential for heavily modified waterbodies and a good ecological status for natural waterbodies (European Parliament and European Council 2000 [EU Water Framework Directive]).

The habitat type 3260 and therefore its target species *C. mercuriale* is mainly threatened by agricultural use, pollution, hydraulic-engineering actions and frequent water management (Ssymank et al. 2021), indicating the necessity of well-conducted management plans for this habitat. However, management of small lotic waterbodies must address specific requirements: when water management is performed too intensive, it is a threat to the biodiversity as well as when water management is not performed or too extensive, so that the habitat conditions are not maintained.

Water management practices as mowing of aquatic vegetation affect invertebrates (Kaenel et al. 1998). For lotic waterbodies, Wright et al. (1992) identified a positive relation between macroinvertebrates and the presence of macrophytes. Especially for Zygoptera (Odonata), submerged macrophytes are crucial (Buchwald 1989; Painter 1998). For fish, a waterbody’s vegetation provides shelter, shade and spawning substrates (Mills 1981; Swales 1982). In case of strong hydraulic forces, reed can slow down the current in the waterbody so submerged macrophytes might also find better conditions to grow along reed (Clark and Samways 1996).

Besides short-term effects, previous studies analyzed the recovery and long-term effect of aquatic vegetation mowing, ranging from 4–6 months (Kaenel et al. 1998) or even 8–11 months (Monahan and Caffrey 1996). A higher negative impact of weed cutting has been analyzed on the abundance of less mobile invertebrate species which may reduce their predator’s abundance in turn due to limited food availability (Kaenel et al. 1998). In long-term, macrophyte communities differ in species diversity and richness, indicating higher values for uncut sites (Baattrup-Pedersen et al. 2002).

Even when there are expected negative effects by water management practices on biodiversity, they do support abundance and diversity of animals as well. Therefore, to maintain high biodiversity in small waterbodies, a certain water management can support (Clarke 2015). Water management prevents sediment accumulation by dredging and regulates vegetation by weeding and mowing, maintaining the hydraulic function (Needelman et al. 2007). Not overgrown and clear of reed sections are favored oviposition sites for Odonata (Buchwald 1989; Painter 1998). *C. mercuriale* prefers a medium vegetation cover for example, avoiding high coverage (Buchwald 1989). Sites with high frequency of emergent vegetation can exhibit a lower macroinvertebrate family richness (Wright et al. 1992). Shade cover, such as by trees, can reduce abundance of Odonata (Remsburg et al. 2008), and ecological practices are known to have a positive influence on the abundance and diversity of amphibians (Maes et al. 2008). Since water vegetation is required but without complete cover and shading of the waterbody (Rouquette and Thompson 2005), threatened species as *C. mercuriale* are dependent on water management concepts that are considering conservation interests. Concepts concerning water management have been developed by many countries (Finér et al. 2018; West Sussex County Council 2018). In Germany, legal requirements to water management are given through the Federal Water Act (Wasserhaushaltsgesetz 2009) and several guidelines from the German Association for Water, Wastewater and Waste [DWA] (e.g. the leaflet from the DWA 2010) and federal states (e.g. Niedersächsischer Landesbetrieb für Wasserwirtschaft, Küsten- und Naturschutz [NLWKN] 2020). Existing water management concepts include seasonality, frequency, practice and equipment. The latter has undergone vast changes, mainly in the last century from manual to motorized, intensifying agricultural practices (Baattrup-Pedersen and Riis 2004; van de Poel and Zehm 2014).

Despite the multiplicity of studies concerning the ecological value and management of small lotic waterbodies, the current practice of water management needs further investigation. The major aim of our study was to investigate the current status of water management especially within the range of the highly, on small lotic waterbody dependent target species *C. mercuriale* to evaluate its biodiversity compatibility and its status to integrate it in the efforts of nature conservation. To gain information about the status in Germany, a questionnaire is used. The questionnaire aims to collect data on reasons, equipment, seasonality, undertaken actions, ecological considerations, and additionally socio-demographic data. The latter is included to understand if there is a correlation between the structure of the executive authority and the water management. The results are then compared with previous studies concerning water management and nature conservation to discuss whether and how the current status meets the recent research on nature conservation and water management interests and requirements. The reconciliation of those two interests aims to improve water management with regard to biodiversity conservation and the goals of the Habitats Directive.

## Methods

### Survey and Data Collection

**Fig. 1:**
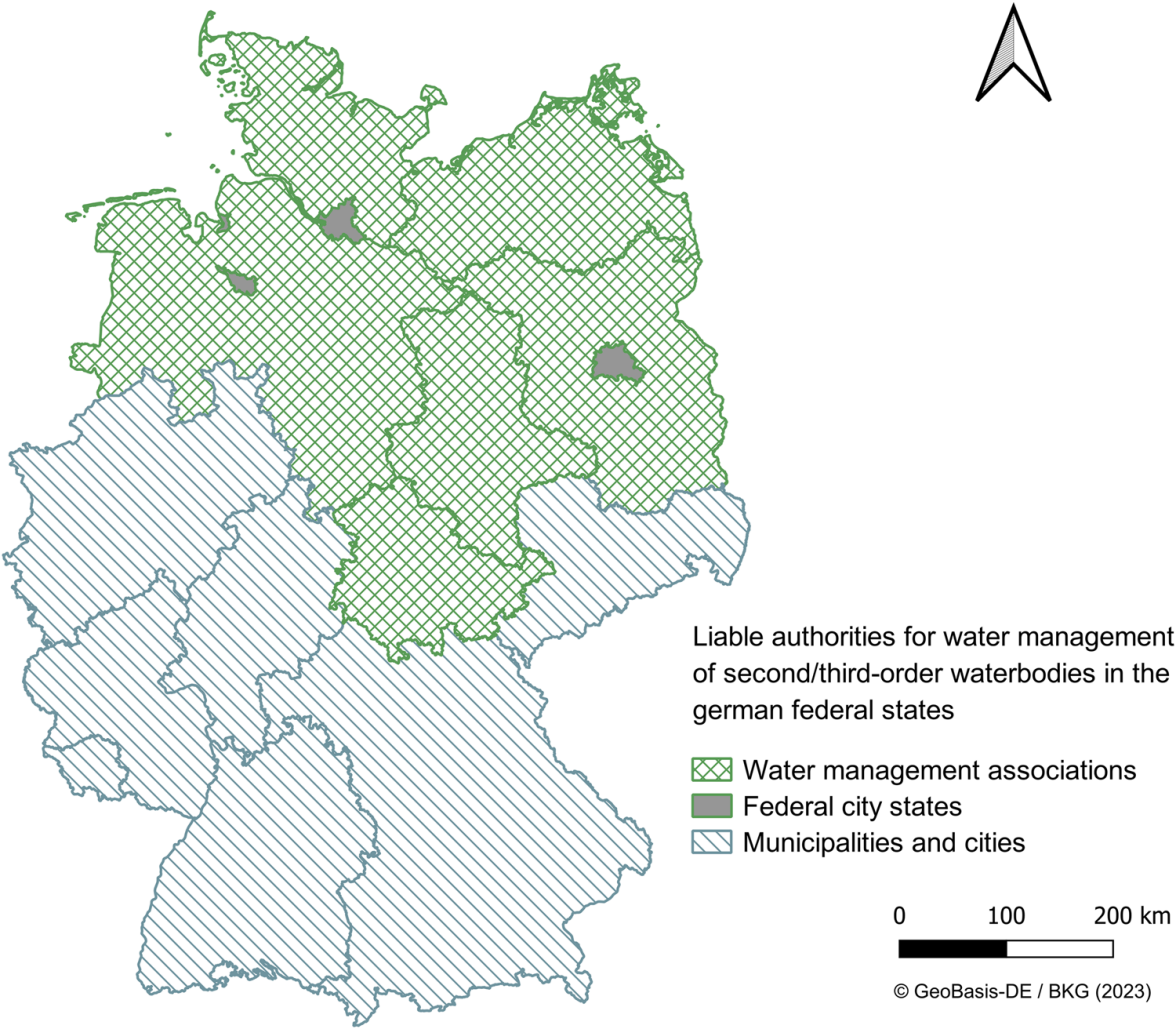
Water management authorities in Germany according to the territories of the federal states. In the federal state marked as green, the territory is divided into local water associations. In the northern federal state Schleswig-Holstein, water associations are usually responsible, occasionally municipalities and cities. For federal states marked as blue, the territory is not divided into water associations. There, governing authorities are responsible, yet they can devote the management responsibility to associations or companies

As *C. mercuriale* is the target species, the study area covers the range of *C. mercuriale* in Germany. The current range of *C. mercuriale* includes 12 out of 16 German federal states. Background data about the range were retrieved from the dragonfly atlas Germany (Brockhaus et al. 2015) and the dragonfly atlases from the federal states or other sources (Baumann et al. 2021; Bayerisches Landesamt für Umwelt 2016; Hill et al. 2011; Hunger et al. 2006; Jäger 2019; Menke et al. 2016; Müller et al. 2018; Trockur et al. 2010; Zimmermann et al. 2005). In addition, the databases ArtenFinder-Portal for Rhineland-Palatinate (Stiftung Natur und Umwelt Rheinland-Pfalz 2020), ASL database Bavaria (Bayerisches Landesamt für Umwelt 2020) and SGL database for Baden-Wuerttemberg (SGL 2020) were consulted. Authorities within the range of *C. mercuriale* in fenlands were not addressed. We identified authorities responsible for management of small lotic waterbodies. Small lotic waterbodies are classified either as second or third order waterbodies, depending on the federal state laws. The management responsibility lies with the owner of the surface waterbodies. Usually, these owners are municipalities in the case of second/third order waterbodies. However, management responsibility can be devolved to associations and companies by the owners. Additionally, owners can be state governments or other governing authorities as well, causing difficulties in identifying responsible authorities. Therefore, the type of authorities can be municipalities, associations, companies, state governments or other governing authorities (Fig. 1).

To gain data on management practices, we prepared a questionnaire. The questionnaire consisted of 21 questions of study interest and one additional for contact details (see Supplementary Information, translated questionnaire). Questions were divided into three sets of questions: (1) socio-demographic (personal) questions, (2) specific questions about water management and (3) questions on the biological background. Socio-demographic (personal) questions involved questions about structure, employees, reasons and relevance for water management, ecological examination and the customer. The reasons and their relevance to the authority included both water management and nature conservation interests. Actions at the water bed/the riparian side/the periphery, equipment, time frame, destination of accruing material due to the actions, scheme type and the working direction, against or with the current, were part of the second set of questions. The equipment question was multiple choice. For water management in bed, several equipment was suggested as e.g. “mowing bucket” and “using a spacer”, followed by an additional free text field for additional equipment. For water management at the shore/bank, equipment was suggested as e.g. “scythe” and “bar mower”, followed by an additional free text field for additional equipment. The whole list of the equipment can be found on page 6 of the questionnaire in the supplement. The shore/bank equipment was combined since according to the feedback of the water management authorities this is performed with the same equipment, i.e. the shore vegetation is managed in the same way as the bank. In addition, participants were asked when actions in water management were performed during the year, according to the seasons. They could specify which actions were performed with additional free text fields.

The biological background and third set of questions involved questions about conservation sites, biological/chemical analysis as for example through fish or nitrate monitoring, knowledge about the occurrence of strongly and especially protected species, knowledge about the damselfly *C. mercuriale* and conservation of species in general. The difference between biological/chemical analysis and the question of the first set of questions of ecological examination is made due to the direct monitoring prior to the water management (ecological examination) which can be done by the authority itself and the scientific monitoring according to for example the WFD (biological/chemical analysis).

Frequency questions were based on water management actions of the leaflet from the DWA (2010). The participants should not answer for their whole area to avoid answers concerning rotating practices at different reaches. This means, that authorities might manage every year reaches, but every year different ones with a rotating system. Therefore, the same reach might be managed only every two or three years, which is the answer that was wanted. In addition, they should differentiate their answer between waterbodies that are managed frequently/far-from-nature and waterbodies that are managed rarely/close-to-nature. According to the received answer during the pre-test (see below), this distinction is made by the authorities.

Questions were designed by categorical variables, mostly allowing multiple choice answers and additional free text fields for additional information if necessary. The participants were asked to answer according to their general water management performance to avoid answers with regard to special and unique water management at reaches of waterbodies (e.g. as part of a conservation project).

A pre-test was carried out by a water management association in Lower Saxony to check the questionnaire for application. After the pre-test, the questionnaire was adapted. Questionnaires were sent by e-mail to the German water management authorities with regard to surface waterbodies.

Subsequently, data collection took place between August 2020 and November 2021. After contacting 181 authorities, 94 questionnaires were distributed, from which 75 (*N* = 181, 41%) were filled out.

### Data Analysis

Statistical analysis was performed with Excel and R (R Core Team 2022). In R, the packages ggplot2 (Wickham 2016), readxl (Wickham and Bryan 2022), reshape2 (Wickham 2007), tidyverse (Wickham et al. 2019) and ggpubr (Kassambara 2020) were used for the diagrams. For significance test, the packages readxl (Wickham and Bryan 2022) and psych (Revelle 2022) were used.

A heat map was created to plot the reasons for water management and their relevance. With the heat map, the number of given answers by the participants can be rated easily by the reader. A bar plot was created to plot the frequency of several actions taking place in the bed as well at the bank. Differently colored bars were used to indicate frequently/far-from-nature and rarely/close-to-nature water management according to the participants’ answers and the number of counts of different answers. Two bar plots were created to plot the number of counts of the used equipment. For better readability, the bar plots were created without the additional equipment answers. Correlations between the usage of equipment and the consideration of species when selecting and applying actions in water management were further analyzed using the Chi-squared test and phi-coefficient due to dichotomous variables. To plot the three parameters seasonality, actions and their count, a three-dimensional diagram was created. A logistic regression was performed to analyze if there is a correlation between the ecological examination and the water management actions in dependence of seasonality. The destinations and their counts of four material accruing due to water management were plotted with four arranged bar plots, one plot for each material (mowing, dug-out, weeding, pruning material). Bars were colored following the three categories “left at site” (red), “disposal” (black) and “utilization” (green) for rating the destinations easily. With a pie chart, the count of the answers “yes, no/not answered, other” to the question of clarifying the occurrence of strongly/especially protected species were plotted to give a clear overview of the majority’s answer. To specify the answer “yes” to the latter, a bar plot was created to demonstrate the main information source.

## Results

**Fig. 2:**
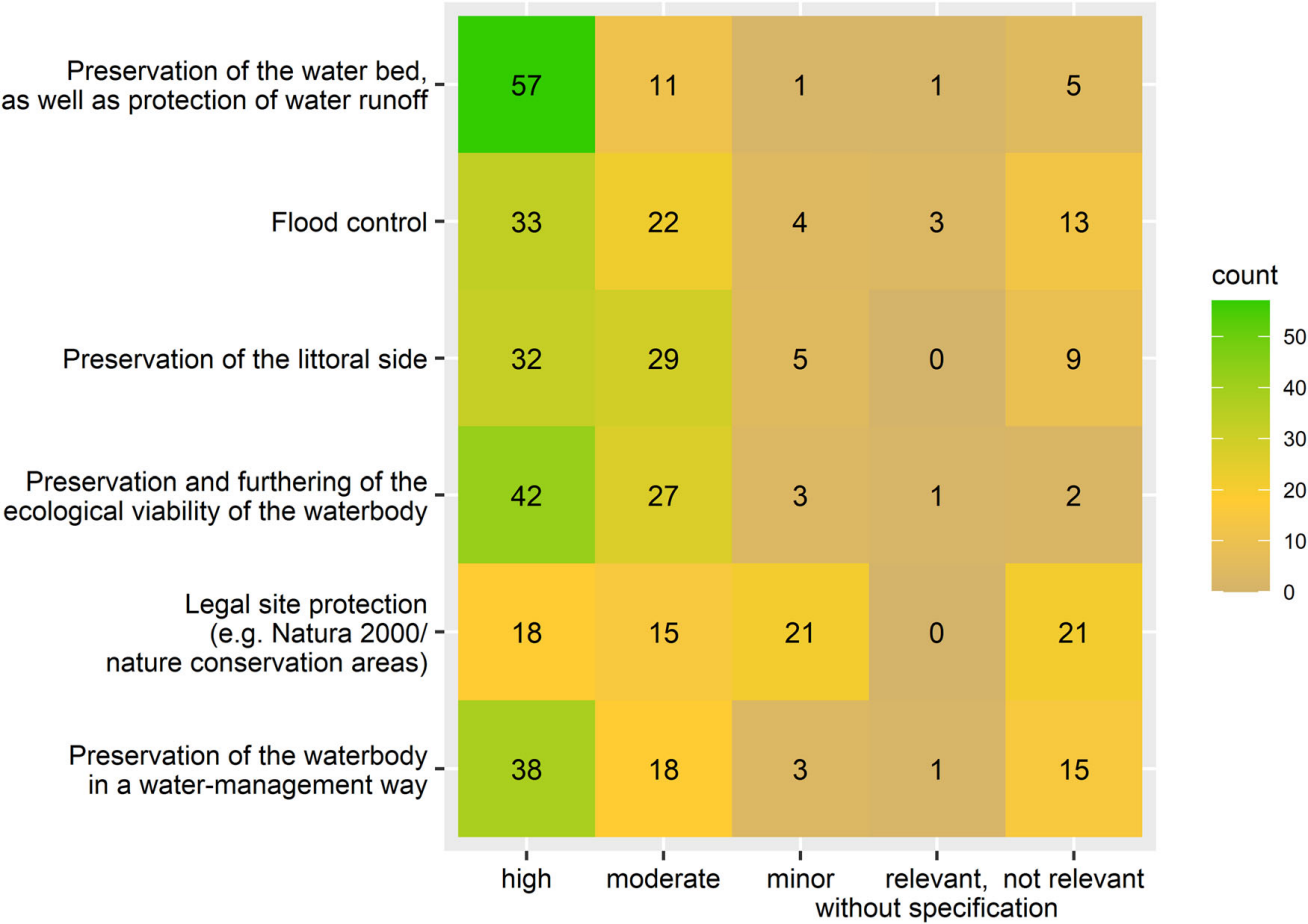
Responses of participants (*N* = 75) concerning reasons for water management and their relevance. Greenish colors indicate higher and greyish colors lower values, i.e. the more greenish the more participants selected the reason with a specific relevance

The analysis of reasons for water management by the authorities showed that a few participants did consider reasons but did not specify the relevance (Fig. 2, “relevant, without specification”). High relevance was conceded to the “preservation of the water bed as well as protection of water runoff” by the majority of the participants (*n* = 57, 76%). Approximately, half of the participants conceded “preservation and furthering of the ecological viability of the waterbody (…)” as well as “preservation of the waterbody in a water-management way” as highly relevant (*n* = 42, 56%, *n* = 38, 51%). “Flood control” and “preservation of the littoral side” were of high relevance for less than half of the participants. “Legal site protection” played a minor part (n(minor) = 21, 28%, n(no relevance) = 21, 28%).

However, the majority (73 out of 75 participants, 97%) stated to consider species conservation interests when selecting and applying actions in water management which was asked by another question. One participant noted that they do not consider species conservation interests yet. Another one did not answer the question. In addition, 53 out of 75 participants (71%) stated to examine the waterbody ecologically before water management, with regard to flora and fauna. Eighteen participants (24%) did not examine the waterbody beforehand, four did not answer the question.

**Fig. 3:**
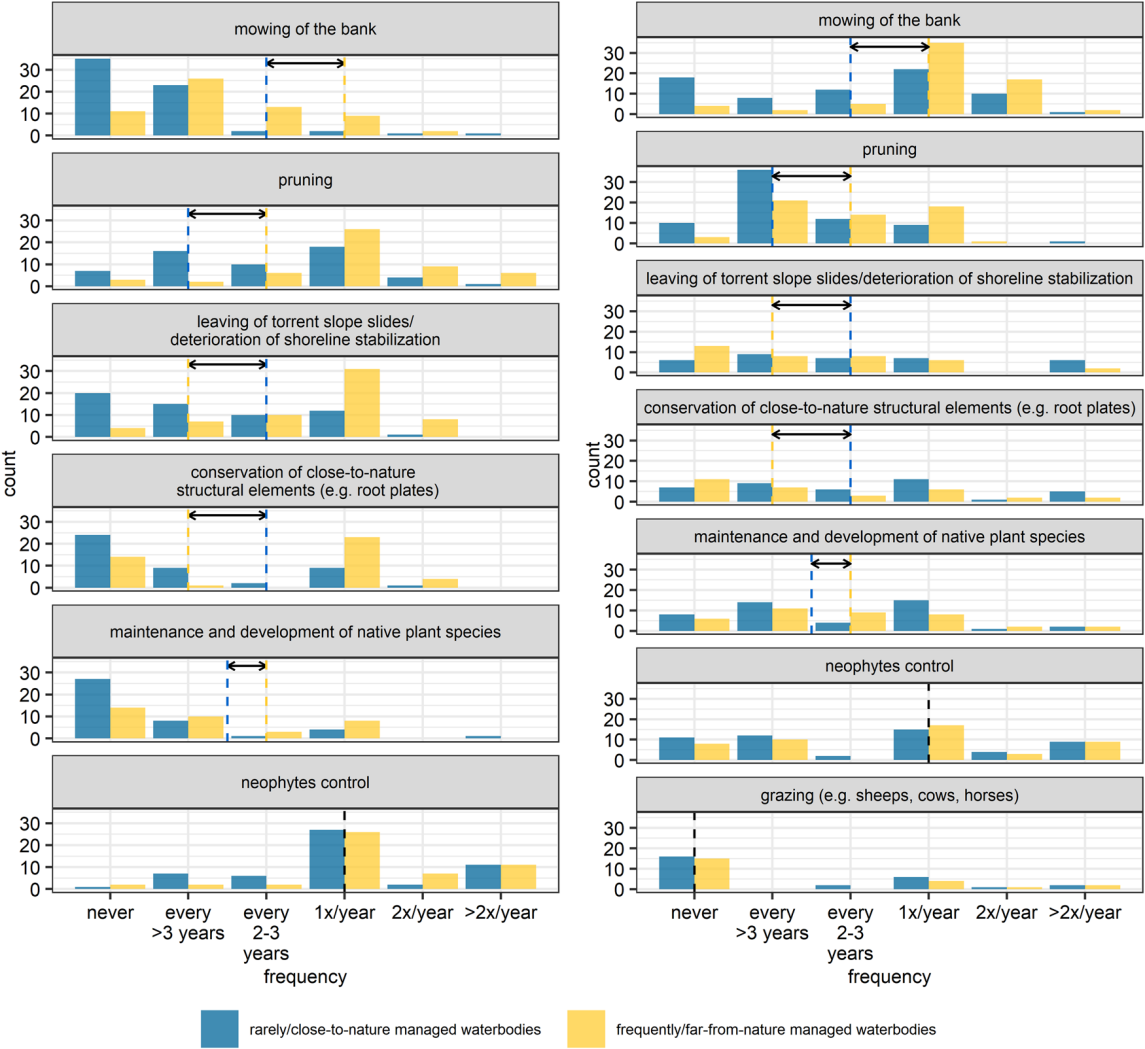
Frequency classification is given with a six-point scale (never, every <3 years, every 2–3 years, 1x/year, 2x/year, >2x/year). The height of the bars indicates the count, the dashed lines the medians and the arrow the difference between those medians of frequently/far-from-nature managed waterbodies and rarely/close-to-nature managed waterbodies. Yellow bars indicate frequently/far-from-nature managed waterbodies, blue bars indicate rarely/close-to-nature managed waterbodies. On the left-hand side, actions (highlighted in gray) taking place in the bed are present. On the right-hand side, actions (highlighted in gray) taking place at the bank are present

The majority of the authorities distinguished between frequently/far-from-nature managed waterbodies and rarely/close-to-nature managed waterbodies. For most actions concerning bed and bank, there were differences between the medians of frequently/far-from-nature and rarely/close-to-nature management (Fig. 3). For removal of litter and refuse (bed) as well as neophytes’ control and grazing (bank), medians were the same. Cleaning of bed was not very frequently performed (median >3 years for frequently/far-from-nature, median never for rarely/close-to-nature respectively). The greatest difference in frequency was present in mowing of water vegetation, followed by weeding. Frequently managed waterbodies were mostly weeded and mowed in bed annually.

**Fig. 4:**
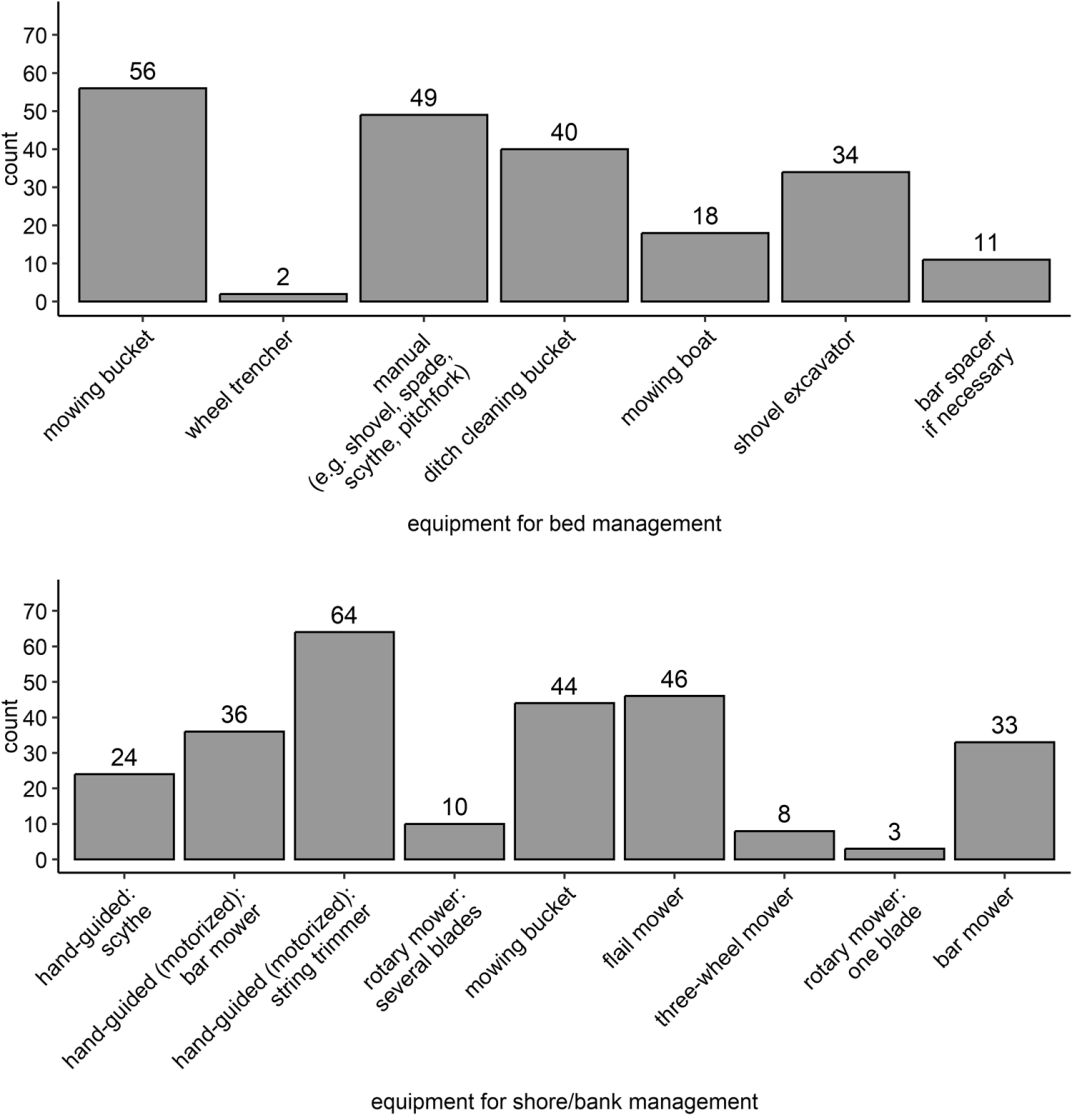
Number of counts of the equipment used in water management by the participants. The question was multiple choice. For water management in bed, equipment was suggested from “mowing bucket” to “using a spacer”, followed by an additional free text field for additional equipment. Therefore, only small numbers are present for the additional equipment which numbers are not shown to receive a better readability. For water management at the shore/bank, equipment was suggested from “scythe” to “bar mower”, followed by an additional free text field for additional equipment which are not shown to receive a better readability

For the equipment in bed (Fig. 4), the mowing bucket was the mostly used equipment (*n* = 56, 75%), followed by manual equipment as shovel or scythe (*n* = 49, 65%), the ditch cleaning bucket (*n* = 40, 53%) and the shovel excavator (*n* = 34, 45%). For the equipment on shore and bank (Fig. 4), string trimmers were used by the majority of the participants (*n* = 64, 85%), followed by flail mower (*n* = 46, 61%), mowing bucket (*n* = 44, 59%), hand-guided (motorized) bar mower (*n* = 36, 48%) and bar mower (*n* = 33, 44%). The most abundant combination of equipment that was selected by 6 of 75 (8%) participants has been “mowing bucket – manual equipment (e.g. shovel, spade, scythe, pitchfork) – ditch cleaning bucket – shovel excavator” for water management in bed. For the shore, the most abundant two combinations of equipment that were selected by each 6 of 75 (8%) participants have been “string trimmer – mowing bucket – flail mower” and “string trimmer – flail mower”. However, there was a scattered picture of equipment combinations, which was demonstrated by the low number of the same choice. Using the trencher was significantly linked to the response that no species conservation interests were regarded to (*r*(1) = 0.7; *p* < 0.001).

**Fig. 5:**
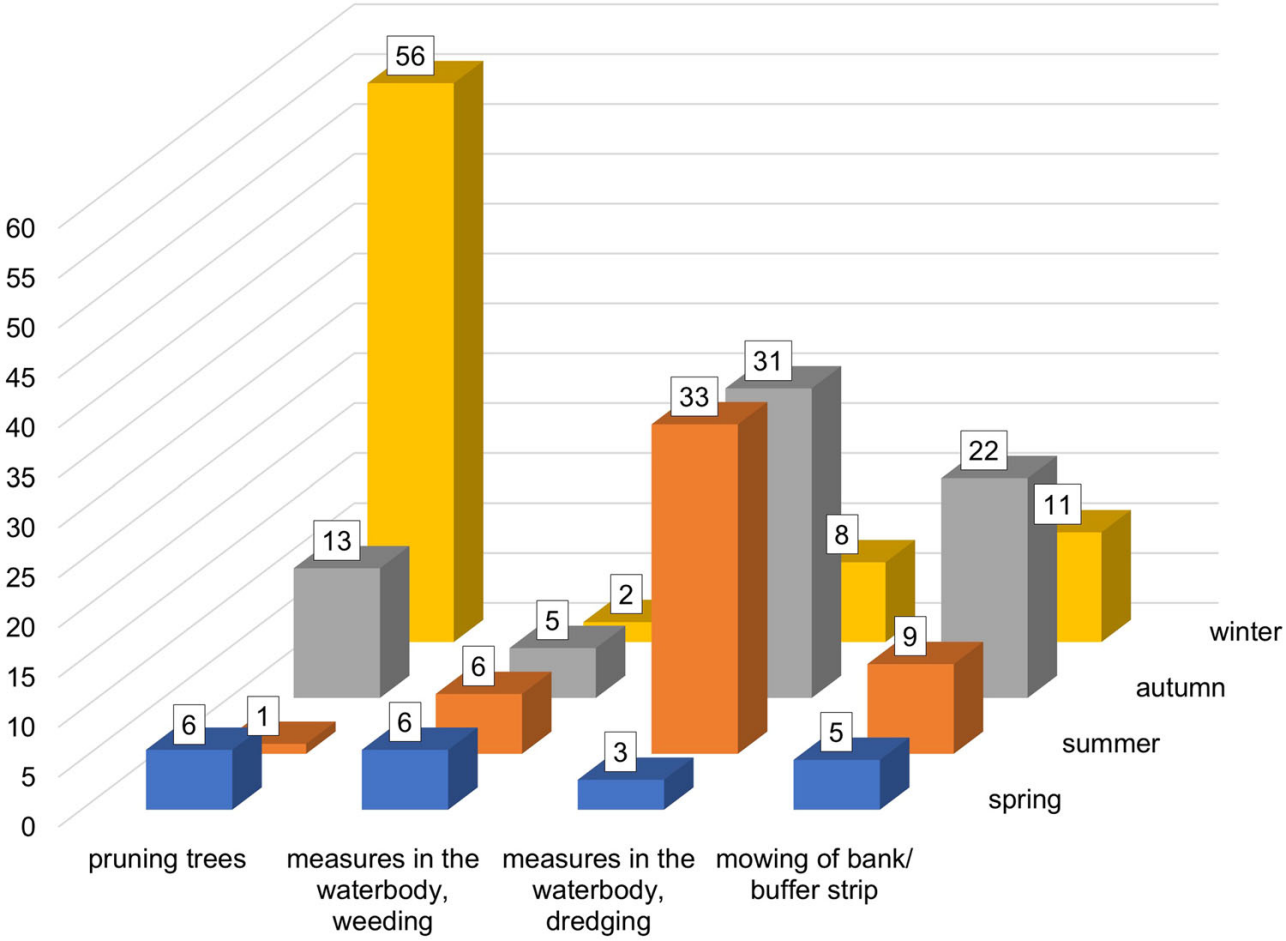
The count of numbers across four main actions in water management are plotted. The different colors indicate the four seasons, the ordinate the count of answers that specific actions are performed in the respective season

Regarding the actions and seasonality, dredging shows small numbers, with its highest in autumn. There were emphases for specific different actions: the emphasis for pruning lied in winter, the emphasis for weeding in summer and autumn and the emphasis for mowing of the bank/buffer strip lied in summer and autumn as well (Fig. 5). The logistic regression model was statistically significant for autumn (X2 (4, *N* = 75) = 0.04, *p* = 0.0055; OR = 9.72, 97.5% [57.97]), indicating that autumn was the preferred season for water management actions when waterbodies were ecologically examined beforehand.

**Fig. 6:**
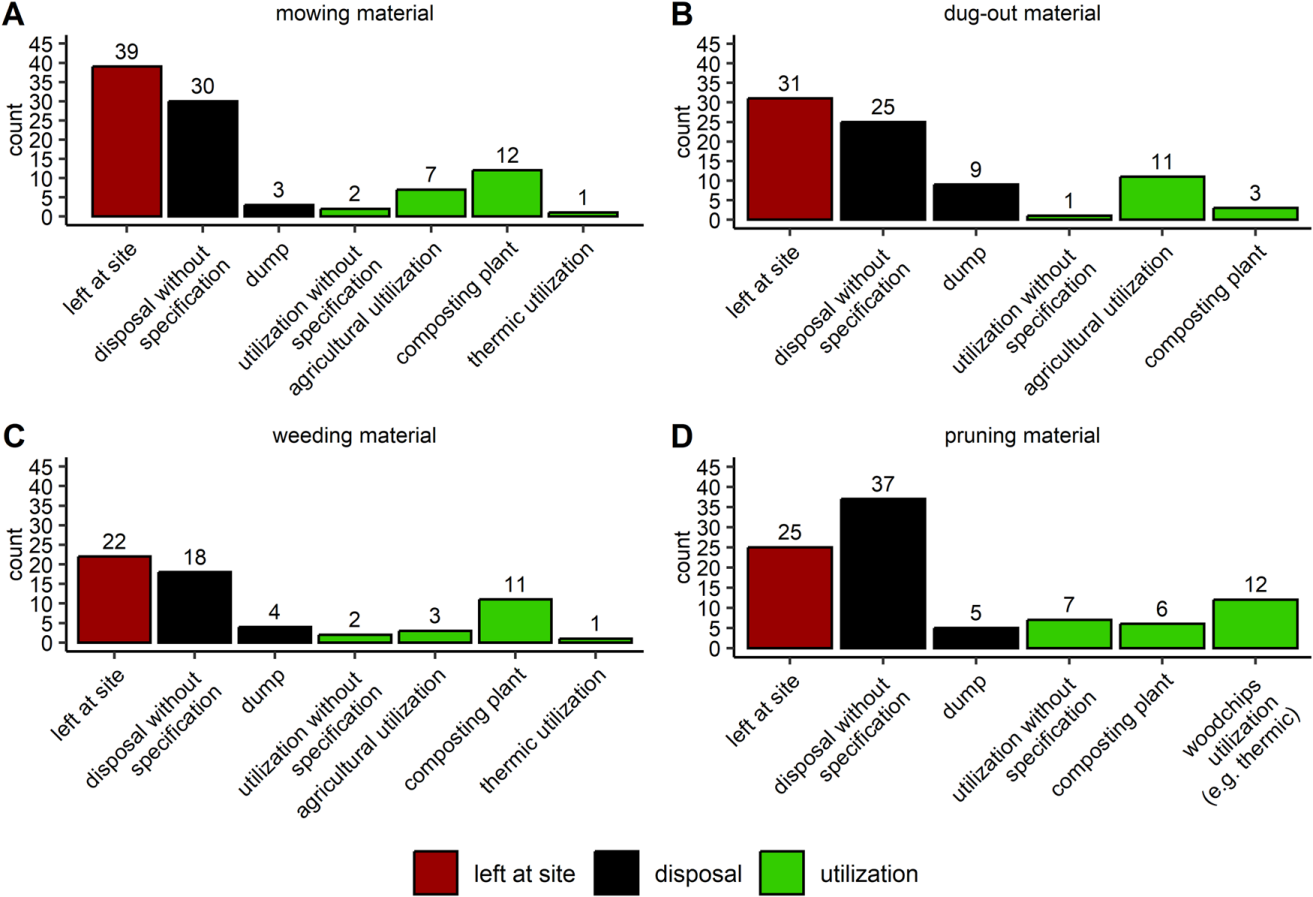
The counts for the destinations of four materials accruing due to water management are plotted. Participants’ answers were categorized into seven (mowing, weeding material) or six (dug-out, pruning material) categorizes, which are additionally highlighted by “left at site” (red), “disposal” (black) and “utilization” (green). Diagram (**A**) shows the destinations of the mowing material, (**B**) the destinations of the dug-out material, (**C**) the destinations of the weeding material and (**D**) the destinations of the pruning material

Mowing, dug-out and weeding material was mostly left at site (Fig. 6). Mowing and weeding material was commonly composted, when its further utilization was specified (*n* = 12, 16%, *n* = 11, 15%). Dug-out material was mostly used for agricultural (*n* = 11, 15%), pruning material for woodchips purpose (*n* = 12, 16%), when its further utilization was specified. A great amount was disposed without specification, which could either mean that the material was utilized or disposed on the dump. When pruning material was left at site, it was often reinstalled into the bed or bank as structural element for example.

**Fig. 7:**
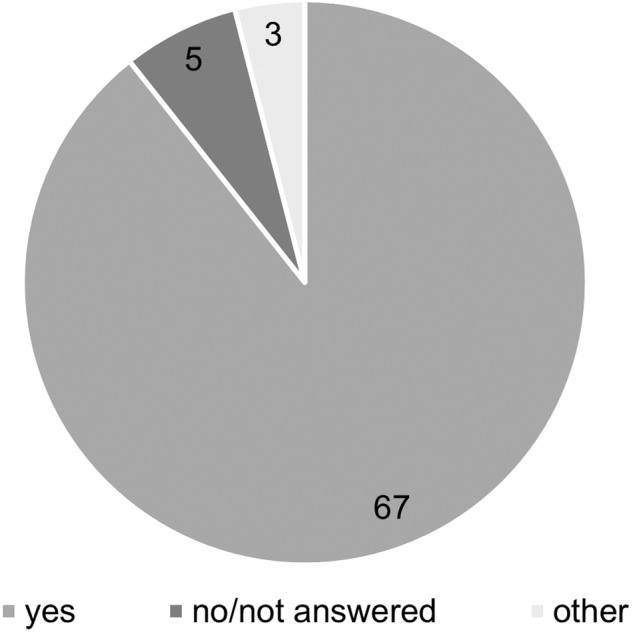
Clarification of the occurrence of strongly/especially protected species when water management is performed at the specific waterbody sites

Conservation interests were clarified by the majority of the participants (89%) in the context of clarification of the occurrence of strongly/especially protected species (Fig. 7). Three participants selected other methods, as it is once the case for the authority being an environmental agency itself, once a biological association which water management is aligned regarding two species of the Habitats Directive (*C. mercuriale, U. crassus*) and once another, not specified clarification. Five participants did not answer the question, which could either indicate that they do not clarify occurrences, or they did not want to answer. Four out of five participants that did not select any answer for clarification, stated additionally to not examine the waterbody ecologically before water management, with regard to flora and fauna.

**Fig. 8:**
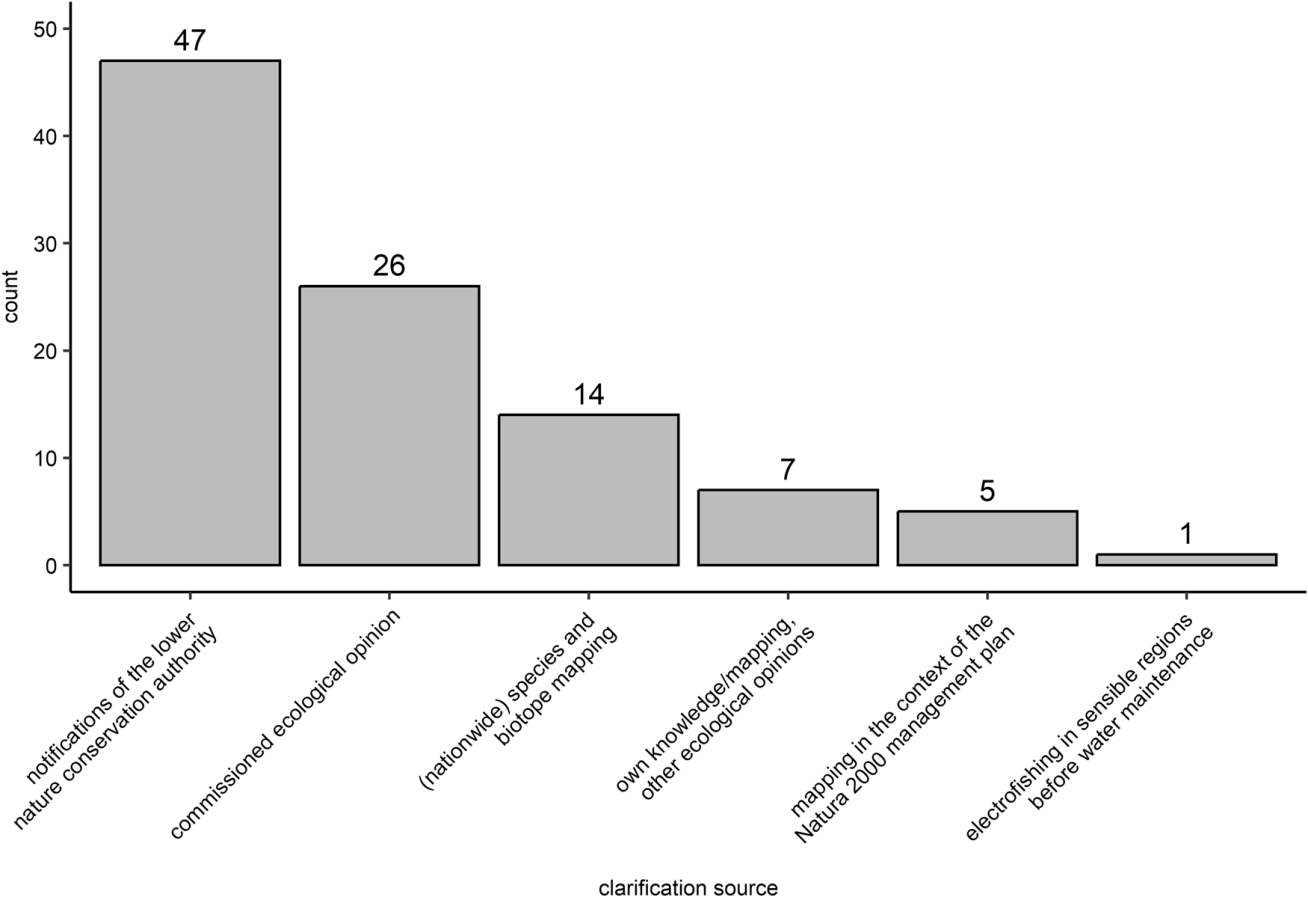
Clarification of the occurrence of strongly/especially protected species, plotted by the count of answers of the participants regarding the type of clarification (*n* = 67, see Fig. 7 “yes”). The first two providers of information were by default, whereas the other four providers have been mentioned by the participants in the “additional free text field” of the question

In the majority, information were retrieved from notifications of the lower nature conservation authority (Fig. 8, 63%). Less relevance did have commissioned ecological opinions. The remaining four clarification possibilities have been not provided by default in the questionnaire. However, fourteen participants stated to clarify occurrences by species and biotope mapping which is often part of monitoring programs of governing authorities.

## Discussion

The present study reveals information about the current water management of small lotic waterbodies. Within the range of the threatened, on small lotic waterbody dependent damselfly *C. mercuriale*, high diversity in taking actions is present. This indicates that water management is sometimes performed with regard to nature conservation interests, but still not throughout the whole studied area. Water management practices are a threat to biodiversity and especially species listed under the Habitats Directive when performed too intensive concerning frequency, equipment, seasonality and applied method which is however often the case according to the study’s results. However, water management is required to maintain the specific habitat conditions. Therefore, water management practices need to be set in context with nature conservation interests on the one hand and with water management interests on the other hand. Eventually it is challenging, but necessary, to address and reconcile both, water management and nature conservation interests to ensure biodiversity and to meet the goals of the Habitats Directive.

### Nature Conservations Interests

The results indicated that nature conservation is of minor interest in water management compared to retaining the hydrological function for example, yet this does not have to be mutually exclusive. Nevertheless, species are impacted by several components in water management, as the seasonality, equipment and frequency. Bączyk et al. (2018) reviewed impacts of in-channel equipment on macroinvertebrates, fish and the general ecological status, with the majority of the reviewed studies indicating negative influences of dredging, macrophyte removal and channel regulation. The reviewing authors suggest to yield precedence to less invasive actions as weed cutting than intense dredging, for instance. In our study, dredging is not frequently performed in the majority of cases. The small number of participants that are practicing dredging more frequently once a year or even more should consider less intense practices, as weeding. Annually weeding and mowing of aquatic vegetation was the median frequency, meeting research outcome. For streams with more than one weed cutting per year, Baattrup-Pedersen et al. (2018) analyzed that the ecological status was moderate to bad. Species diversity and composition decline with the named frequent practices (Baattrup-Pedersen et al. 2002). According to van Strien et al. (1991), optimum species richness of ditch bank vegetation occurs with cleaning once every 2–3 years. Therefore, annually weeding and mowing of vegetation in bed should be the bottom line in frequency, when there is no less maintenance frequency possible according to the duly runoff.

Technical inventions for equipment increased to match the need for increasing yield. With the motorization of to date existing manually applied mechanical machines, greater and faster machines became popular. This circumstance resulted in increasing negative impacts on species. Aldridge (2000) analyzed that 3–23% of a mussel population was found in the spoil after dredging, whereas weed boats did damage a small number, but did not remove any mussels. In our study, dredging is performed with a ditch cleaning bucket or shovel excavator in the majority of cases, yet not frequently. For perennial ditches, dredging with the wheel trencher is forbidden according to the German Federal Nature Conservation Act since 2009, if a significant damage is expected for the ecosystem and especially the fauna (Bundesregierung 2009/18.08.2021 [BNatSchG]). Our results reflect the legal landscape: the use of the wheel trencher is significantly correlated to no consideration of conservation species’ interests. If they would be considered, damage would be expected since small lotic waterbodies exhibit a variety of (rare) species. In the case of Natura 2000 sites with the EU habitat 3260, this also reflects a lack in legal implementation and management as most of these species are part of the habitats typical species and habitat deterioration is to prevent in designated sites according to Art. 6 (2) Habitats Directive. However even official monitoring schemes under the Habitats Directive have this shortcoming as typical animal species are mostly not monitored and missing the German agreed monitoring assessment schemes (Bundesamt für Naturschutz and Bund-Länder-Arbeitskreis FFH-Monitoring & Berichtspflicht 2017).

The mowing bucket is the mostly used equipment in water management of the bed in the present study, followed by manual equipment as shovel or scythe. Monahan and Caffrey (1996) analyzed that the mowing bucket does have a great impact on macroinvertebrate numbers, causing the greatest reduction compared to three other weeding techniques (in their study: harvester, chemical herbicide dichlobenil, Wilder boat). Conventional restricted hydraulic possibilities of swiveling and an often-obstructed sight leads to interference with bed and soil, causing erosion and similar ecological impacts as dredging. Therefore, the mowing bucket is only recommended when applied 10 cm over the bed, e.g. by using a spacer (DVWK 1992). Equipment with a spacer is used by approximately 15% of the participants. Prospectively, there are new inventions concerning the mowing bucket, as a fully pivotable, ecologically working hydraulic small mowing bucket, mowing only a narrow channel (Tschöpe 2020).

Since large numbers and biomass of invertebrates are removed with the cut weed (Dawson et al. 1991), it is recommended to leave the material at site for a short time to reduce these impacts. Fauna can then escape into adjacent biotopes and the transportation of dry matter is facilitated (DVWK 1992). A small amount of the participants additionally states to have left the material at site for short before removing it, even when this process is more time-consuming and expensive since it includes two operations.

The scythe has smaller impacts on physical habitat quality than the mowing boat, whose applied method differs itself between a less intense shallow method and a deep one (Rasmussen et al. 2021). In addition, mowing boats with scythe chains or triangular scythes interfere with soil, whereas T-front mowers above the bed spare fauna and bed (DVWK 1992).

Regarding equipment used for the bank and shore, various studies exist concerning the impacts of mowing on different animals (e.g. Classen et al. 1996; Grendelmeier 2011; Hemmann et al. 1987; Humbert et al. 2010). van de Poel and Zehm (2014) reviewed tendencies and effects on animal species which often are injured or killed by mowing equipment. The reviewing authors state that rotary machines do have a greater impact than cutting machines, since rotary machines work in a greater working space with a higher velocity. Consequently, the greater impact of rotary machines on the fauna is opposite to their efficiency. In our study, rotary machines are used by the majority (e.g. hand-guided string trimmer 85%, flail mower 61%). Analyzed for roadside verges, the flail mulcher has a great negative impact on arthropods, with the highest loss of 87% for Lepidoptera (Steidle et al. 2022). From an efficiency perspective, the flail mulcher can remove smaller groves, directly mulches the cut grass and leaves it at site, saving the procedure of transportation. However, the ecological impact is not neglectable as well as the availability of excess nutrients which fasten up regrowth. Less intense mowing techniques than rotary machines are yet used by several participants. Those include the hand-guided bar mower (48% of the participants), bar mower (44%) and scythe (32%). The mowing bucket (approx. 59%) works with the bar mower technique as well but can affect the soil as it is discussed before.

To our knowledge, research studies advise to refrain water management practices in the water bed in spring, with regard to both flora and fauna (e.g. Kaenel et al. 1998; Westlake and Dawson 1988). In the present study, weeding and dredging practices are performed by the participants’ minority in spring, following the research recommendations. Weeding and dredging in spring and (early) summer affect fish, breeding birds and mussels (Aldridge 2000). The Roach *Rutilus rutilus* is spawning in spring and early summer for example, with eggs on vegetation close to the surface, leading to the threat by water management of falling water levels (Mills 1981). Disturbing water management does have a negative impact on amphibian spawn and larvae in spring and early summer, when they are present (Leiders and Röske 1996; Twisk et al. 2000). Concerning vegetation diversity, weeding in autumn leaves diaspores of annual vegetation, whereas a great amount of competitive perennial vegetation is removed (Garniel 2012). Furthermore, amphibians do hibernate in soil or under moss in winter (Holenweg and Reyer 2000), so that water management in bed represents a threat in winter (Leiders and Röske 1996). However, dredging is still performed by approximately 15% in winter, weeding by around 11% respectively. The majority does perform weeding, dredging and mowing in summer and autumn, which mostly avoids reproduction, spawning and hibernation times. Pruning is performed by the distinct majority in winter, which matches the legally given times of pruning outside forests (exceptional is maintenance pruning) between 1st of September and 28th of February in Germany (Bundesregierung 2009/18.08.2021 [BNatSchG]). There are similar effects of bank and meadow mowing (Leiders and Röske 1996). Meadow floral species can be mown twice a year, whereas tall herbaceous vegetation and reed should be mown only once in autumn (Leiders and Röske 1996). For *C. mercuriale*, observations by Röske (1995) even indicate that already regrowing bank areas are more preferred than uncut ones as well as recently mown more or less bare bank areas. However, working techniques do mostly allow only one operation, both in bed and shore/bank actions, as it is more cost-efficient and requires less staff.

Emphases of weeding and mowing of bank/buffer strip in summer and autumn indicate that the majority of the water management authorities is already avoiding seasonal conservation conflicts during spring and winter. Research tends to result in preferring late summer and autumn for water management actions, especially for waterbed actions. Our study indicates that authorities might prefer autumn as water management season when waterbodies are ecologically examined beforehand. However, there are a few participants practicing weeding in spring and winter, disturbing fauna during these seasons.

### Water Management Interests

Besides nature conservation interests, water management interests must be addressed as well to extend the point of view when reconciling both interests. Increasing aquatic vegetation growth can cause failure of the waterbody’s drainage function (Aldridge 2000), which is reflected by the participants’ answers: The highest and most rated reason for water management was the preservation of the water bed as well as the protection of water runoff. Traditional reasons for water management in agricultural land, such as the mitigation of floods (Bączyk et al. 2018), were highly relevant for 44% of the participants. The results indicate that nature conservation interests as legal site protection indeed hold a minor part for the reasons of water management, whereas water management interests as mitigation of flood are of higher relevance. However, the majority of the water management authorities might consider some sort of ecological significance since they distinguished between frequently/far-from-nature and rarely/close-to-nature managed waterbodies. Even when management actions can differ due to site-specific, hydrological properties, general tendencies demonstrated that there is a great difference in frequencies when mowing of water vegetation and weeding are performed. Cleaning of bed as dredging is not frequently performed, yet it sometimes has to be carried out when silt accumulation takes place.

Challenging the failure of the waterbody’s drainage function due to aquatic vegetation, late summer is suggested due to a decelerated regrowth (Baattrup-Pedersen et al. 2018). Westlake and Dawson (1988) even analyzed that autumn is the preferable season for weeding, which reduces plant biomass in contrast to weeding in spring, keeping water levels low, preventing flooding and avoiding late summer cuts. According to our results, weeding is already performed mostly in summer and autumn.

Regarding further challenges in water management, a participant stated (authors’ translation): “The use (in the worst-case disposal, in the best-case utilization) of the landscape management material is one of the greatest challenges.” This is demonstrated by our study as well. Accruing material in water management is mostly left at site or just disposed without utilization. Pruning material is used by 16% of the participants for thermic utilization. A high amount of the mowing and weeding material is left at site, leading to the reflow of nutrients of rotting vegetation into the waterbody, promoting vegetation growth as nutrient-rich fertilizer (Moeller and Zehnsdorf 2017). In addition, left at site mowing/weeding material can float, drift and cause problems in the runoff at sites further downstream as well as causing bleak areas due to inhibition of vegetation growth (1992). As a result of eutrophication induced by left at site material, changes in bank vegetation composition are expected to nutrient indicator plants as *Urtica dioica* (DVWK 1992). The flail mower combines cutting und mulching, so that the mowing material is left at the site.

Accruing material in water management has a great potential to be used, even when the current numbers did not reflect the possibilities. Legal regulations might be complicated to understand, so that further consideration of utilization is time-consuming. In addition, transportation and disposal are expensive in regard to other water management practices. It is simple to save costs by letting the material at site. However, vegetation growth is then promoted, leading to more frequent water management – so it is questionable, if this saves costs after all.

### Reconciliation of Both Interests

Communication is crucial to reconcile water management and conservation interests. To address ecosystem services and the loss of biodiversity (Young et al. 2014), knowledge exchange is essential for effective environmental management (Fazey et al. 2013). However, there are deficiencies in communication. Exchange often takes place within or between expert associations (Riecken et al. 2020), so that scientific transfer happens “behind closed doors” and does not reach practitioners. The dealing with the wheel trencher reflects the poor communication between science, policy and practice: The research already pointed out strong negative ecological effects of the wheel trencher for several decades (Leiders and Röske 1996). However, it was prohibited nationwide not before 2009.

Since primary scientific literature is infrequently accessed due to time consuming search and reading (Pullin et al. 2004), research outcome does not always reach practice. Poor communication leads to reliance on the current status, continuing with the practice that has always been performed (Pullin et al. 2004). On top negative changes due to more intensive practices or “better” modern equipment are often completely neglected or ignored. However, interest in communication and the provision of recommendations are present. In our study, several participants did ask for outcome and practical implementation advices (recommendation for action) as important need, even though there is a variety of existing water management concepts. That indicates poor knowledge exchange as well.

The inclusion of all stakeholders and agreements made in water management are time-consuming, yet it is necessary to consider both, hydrological and ecological needs in management practices. Four out of five participants who did not select any answer for clarification of the occurrence of especially/strongly protected species at managed waterbodies stated to not examine the waterbody ecologically before water management, with regard to flora and fauna. Nevertheless, this is true for the minority, as we hypothesize that no clarification in any way leads to water management considering no or barely ecological purposes, even when approximately 97% of the participants state to consider species conservation when selecting and applying actions in water management. Keeping that in mind, a good ecological potential or status are however set goals in the EU Water Framework Directive, as well as a favorable conservation status of the Habitats Directive when the species live in an EU protected flowing water habitat (3260).

Municipalities and the corresponding department responsible for water management do often perform several other tasks as road and green structure maintenance as well, struggling with time resources. The implementation of water associations according to river basins/catchments is one possible solution, replacing municipalities as water management authorities and centralize water management tasks. Water associations are already present in half of the federal states in Germany, having responsibility for water management. Specific tasks as water management facilitate a holistic approach including agreements with stakeholders (e.g. the lower nature conservation authority). The lower nature conservation authority communicates, in turn, with (monitoring) scientists and seeking exchange with scientific associations. It already has major significance with regard to the clarification of the occurrence of especially/strongly protected species at managed waterbodies, especially since it represents the link between policy and science.

To reconcile both interests, the main challenge is to exchange knowledge, to map stakeholders and to work altogether on management plans. Water management practices can have positive effects on abundance and diversity of species, as it is the case for Odonata as the damselfly *C. mercuriale*. This can result in collaboration rather than working alone or in the worst-case reproaching and working against each other – especially since there is a great ecological potential in water management, and interests in reducing climate change effects also usually coincide.

### Consequences for the Habitats Directive

Improved communication contributes to the Habitats Directive by implementing water management plans for conservation areas and beyond. Knowledge exchange about occurrence of species listed under the Habitats Directive as *C. mercuriale*, *U. crassus* or *M. fossilis* attracts attention and can promote change in the status quo of water management to an ecological point of view. Typical species of the protected habitat type 3260 under the Habitats Directive are threatened i.a due to frequent water management (Ssymank et al. 2021).

The habitat type 3260 provides habitats for fish, invertebrates, birds, amphibians, reptiles and mammals, as well as food availability for birds, bats and other mammals, indicating the importance of considering consequences when performing water management. Some habitat requirements of protected species are in conflict with the general goal of wood growth, e.g. the development from habitat type 3260 to habitat type 91E0 (Alluvial forests with *Alnus glutinosa* and *Fraxinus excelsior* (Alno-Padion, Alnion incanae, Salicion albae)). Wood growth is a threat to species that are dependent on a partly open water surface as the protected damselfly *C. mercuriale*. Therefore, communication and knowledge exchange are crucial to reach a favorable conservation status for protected species.

According to the state of nature report 2013–2018, legal site protection actions mostly concentrate on keeping the status quo, avoiding a decline rather than improving actively the conservation status (European Environment Agency 2020). Existing concepts, such as these in water management, should yet be reviewed according to their consideration of positive and negative effects on biodiversity. By exchanging knowledge of both sites, practice and research, adaptions in water management could be made, challenging improvements besides stabilization actions.

By managing small lotic waterbodies in an ecological feasible way in the agricultural landscape, those habitats contribute to the European Biodiversity Strategy for 2030 where “at least 10% of agricultural area is under high-diversity landscape features” (European Commission, Directorate-General for Environment 2021). In addition, water management considering species listed under the Habitats Directive contributes to the key commitment of the EU Biodiversity Strategy that “at least 30% of those not already in favorable conservation status reach that category or show a positive trend” by the year 2030 (European Commission, Directorate-General for Environment 2021). Improved communication, collaboration and consideration of conservation key goals can result in a higher relevance of conservation interests than it is the case for the current status of water management.

## Conclusions and Limitations

The present study reveals the current status of water management and knowledge about several aspects of water management is gained which has not yet been available to this extend. These are necessary to improve water management with regard to nature conservation of small lotic waterbodies. However, this research has some limitations that should be considered. First, the application area is limited to Germany. In addition, the detection of responsible authorities turned out to be challenging, so that there is a small possibility that not all the authorities were contacted. During the survey, there were a few participants who answered that they only practice close-to-nature methods, so there might be smaller deviations.

In conclusion, the present study demonstrates that challenges in water management mainly result in the balancing act between water management interests (i.e. especially the preservation of the water bed/protection of water runoff) and conservation interests. It demonstrates that the implementation of available knowledge in practice is still a challenge, even when ecological performance due to the goal of a good ecological potential/status according to the Water Framework Directive has given increasing priority (Bączyk et al. 2018; European Parliament and European Council 2000 [EU Water Framework Directive]). However, small lotic waterbodies need special attention due to their function as biodiversity hotspots of vast amount.

There are improvements that can easily be adapted, as it is the case for communication, seasonality, choice of equipment and frequency, which exhibit a multiplicity of studies. However, there are still challenging factors in water management as the handling with accruing material, which need further notice. Yet, water management, performed in close communion with nature, can contribute to nature conservation, especially in the context of the conservation status of protected species under the Habitats Directive.

### Supplementary Information

SUPPLEMENTARY INFORMATION 1
