# Supplementary material for: Current Water Management of Small Lotic Waterbodies in the Context of Nature Conservation in Germany
Source: Environ Manage. 2023 Nov 4;73(1):162–76. doi: 10.1007/s00267-023-01904-y (PMC10786963; doi:10.1007/s00267-023-01904-y)
Supplement: Supplementary file 1 — SUPPLEMENTARY INFORMATION 1 [file 267_2023_1904_MOESM1_ESM.pdf]

SUPPLEMENTARY INFORMATION 1

Questionnaire concerning water management as part of a doctoral project on the biology and protection of damselflies at waterbodies

Dear Sir or Madam,

Thank you for taking the time to complete this questionnaire. Please fill it out as completely and precisely as possible. Due to the subject matter, there are no "right/wrong" answers. With your statements you provide an important contribution to the compatibility of necessary water management actions and nature conservation concerns.

The background: In the context of my PhD project at the Federal Agency for Nature Conservation in cooperation with the University of Education Karlsruhe, I am dealing with the topic of water management and protection concepts of the damselfly *Coenagrion mercuriale*. The project is about the performed actions of water management, and, as already mentioned above, the compatibility with conservation concerns. In this regard, I am interested in performed actions, the reasons as well as information about the waterbodies. The main goal is to develop a guideline for water management in the context of nature conservation that is not only based on theory, but that is practical and can actually be implemented.

The use of your statements for my research requires your consent. Your data will only be treated/published anonymously and confidentially to ensure data protection. Furthermore, your data will not be passed on to third parties. Participation is voluntary. Of course, the processing of the questionnaire can be interrupted at any time.

Thank you for your time and for completing the questionnaire. The questionnaire should take about 20-30 minutes to complete.

If you agree with the above, please sign here:

________________________ ____

Place, date Signature

If you have any further questions, even after filling it out, please feel free to contact me.

Isabelle Immerschitt
Federal Agency for Nature Conservation
Personal E-Mail address: isabelle.immerschitt@t-online.de

PERSONAL QUESTIONS

Question 1:
Which working structure do you belong to?

- ☐ Independent company (e.g. limited liability company, planning office): ____________________
- ☐ Governing authority (e.g. building authority of the municipality): ____________________
- ☐ Association (e.g. landscape maintenance/biological station):____________________
- ☐ Other: ____________________

Question 2:
How many employees are in your working environment?

- ☐ < 5
- ☐ 5-10
- ☐ 11-15
- ☐ 16-30
- ☐ > 30

<u>Question 3:</u>

What are the reasons for performing water management and how relevant are they?

– multiple responses are possible –

	relevance		
	high	moderate	minor
☐ Preservation of the water bed, as well as protection of water runoff (e.g. drainage ditches, road side ditches)	☐	☐	☐
☐ Flood control (Prevention of flood damage)	☐	☐	☐
☐ Preservation of the littoral site, especially due to preservation and new planting of a riparian vegetation appropriate to the site, keeping the banks free for water runoff	☐	☐	☐
☐ Preservation and furthering of the ecological viability of the waterbody, especially as habitat for fauna and flora	☐	☐	☐
☐ Legal site protection (e.g. Natura 2000/nature conservation areas)	☐	☐	☐
☐ Preservation of the waterbody in a water-management way (with regard to the discharge or retention of water, bedload, suspended solids and ice)	☐	☐	☐
☐ Other: ____	☐	☐	☐

<u>Question 4:</u>

Is an (ecological) examination of the flora and fauna of the waterbodies carried out by you or a third party (also "look into the waterbody": recording of *Elodea spp., Iris spp....*)?

☐ Yes

☐ Yes, with regard to certain species:

☐ No

SPECIFIC QUESTIONS ABOUT WATER MANAGEMENT

Note on questions 5-7: For the frequency of specific water management actions, tick the frequency that applies to most of the waterbody reaches you manage. A distinction is made between "rarely/close-to-nature" and "frequently/far-from-nature" water management. Use your best judgement to decide which reaches you consider to be managed in a close-to-nature or far-from-nature way. Example: A waterbody reach that is only managed to achieve a good ecological status may be managed less frequently than one that is managed to maintain continuity.

Depending on the size of the area of responsibility, a bed may be cleared every year, only from different waterbodies. However, these questions and answers are intended to be ticked in relation to individual reaches of waterbodies and not the entire area of responsibility.

Question 5:

Which actions on the bed are carried out by your company/your authority/ your association on a single reach of waterbody (not related to the entire area) and how often?

– Distinction between reaches with rarely/close-to-nature management and frequently/far-from-nature management –

	rarely/close-to-nature management: never	every >3 years	every 2-3 years	1x /year	2x /year	>2x /year	frequently/far-from-nature management: never	every >3 years	every 2-3 years	1x /year	2x /year	>2x /year
☐ cleaning of bed	☐	☐	☐	☐	☐	☐	☐	☐	☐	☐	☐	☐
☐ clearing of local runoff obstacles (e.g. dead wood)	☐	☐	☐	☐	☐	☐	☐	☐	☐	☐	☐	☐
☐ weeding	☐	☐	☐	☐	☐	☐	☐	☐	☐	☐	☐	☐
☐ mowing of water vegetation (e.g. with mowing boat)	☐	☐	☐	☐	☐	☐	☐	☐	☐	☐	☐	☐
☐ stabilization measures of bed: which?	☐	☐	☐	☐	☐	☐	☐	☐	☐	☐	☐	☐
☐ removal of litter and refuse	☐	☐	☐	☐	☐	☐	☐	☐	☐	☐	☐	☐
☐ other	☐	☐	☐	☐	☐	☐	☐	☐	☐	☐	☐	☐

Question 6:

Which actions on the bank are carried out by your company/your authority/ your association on a single reach of waterbody (not related to the entire area) and how often?

– Distinction between reaches with rarely/close-to-nature management and frequently/far-from-nature management –

	**rarely/close-to-nature management** frequency						**frequently/far-from-nature management** frequency					
	never	every >3 years	every 2-3 years	1x /year	2x /year	>2x /year	never	every >3 years	every 2-3 years	1x /year	2x /year	>2x /year
☐ mowing of the bank	☐	☐	☐	☐	☐	☐	☐	☐	☐	☐	☐	☐
☐ pruning	☐	☐	☐	☐	☐	☐	☐	☐	☐	☐	☐	☐
☐ leaving of torrent slope slides/deterioration of shoreline stabilization	☐	☐	☐	☐	☐	☐	☐	☐	☐	☐	☐	☐
☐ conservation of close-to-nature structural elements (e.g. root plates)	☐	☐	☐	☐	☐	☐	☐	☐	☐	☐	☐	☐
☐ maintenance and development of native plant species	☐	☐	☐	☐	☐	☐	☐	☐	☐	☐	☐	☐
☐ neophytes control	☐	☐	☐	☐	☐	☐	☐	☐	☐	☐	☐	☐
☐ grazing (e.g. sheeps, cows, horses), p.r.n. by third parties	☐	☐	☐	☐	☐	☐	☐	☐	☐	☐	☐	☐
☐ other: ______________________________	☐	☐	☐	☐	☐	☐	☐	☐	☐	☐	☐	☐

Question 7:

Which actions on the environment are carried out by your company/your authority/ your association on a single reach of waterbody (not related to the entire area) and how often?

– Distinction between reaches with rarely/close-to-nature management and frequently/far-from-nature management –

	**rarely/close-to-nature management** frequency						**frequently/far-from-nature management** frequency					
	never	every >3 years	every 2-3 years	1x /year	2x /year	>2x /year	never	every >3 years	every 2-3 years	1x /year	2x /year	>2x /year
☐ maintaining a riparian strip	☐	☐	☐	☐	☐	☐	☐	☐	☐	☐	☐	☐
☐ extensification of the use	☐	☐	☐	☐	☐	☐	☐	☐	☐	☐	☐	☐
☐ development/creation of cutoff meander/trough/side channels/floodplain	☐	☐	☐	☐	☐	☐	☐	☐	☐	☐	☐	☐
☐ other: ______________________________	☐	☐	☐	☐	☐	☐	☐	☐	☐	☐	☐	☐

Question 8:

Which equipment is used for water management?

– Multiple responses are possible –

Actions in the bed

- ☐ mowing bucket
- ☐ wheel trencher
- ☐ manual (e.g. shovel, spade, scythe, pitchfork)
- ☐ ditch cleaning bucket
- ☐ mowing boat
- ☐ shovel excavator
- ☐ above ticked off equipment: bar spacer (if necessary)
- ☐ other: ____

Actions on the bank

- ☐ hand-guided: scythe
- ☐ hand-guided (motorized): bar mower
- ☐ hand-guided (motorized): string trimmer
- ☐ rotary mower: several blades
- ☐ mowing bucket
- ☐ flail mower
- ☐ three-wheel mower
- ☐ rotary mower: one blade
- ☐ bar mower
- ☐ other: ____

Question 9:

When are the water management actions performed?

– Multiple responses are possible –

If certain methods essentially (do not) take place at a certain time of the year, feel free to note this next to the answer (e.g., no cleaning of the bed in the fall; only pruning in the winter).

☐ spring: ____

☐ summer: ____

☐ autumn: ____

☐ winter: ____

Question 10:

How is the destination of the accruing material from the actions regulated? Briefly explain

– Multiple responses are possible –

☐ mowing material: ____

☐ dug-out material: ____

☐ weeding material: ____

☐ pruning material: ____

☐ other: ____

Question 11:

What type of water management in terms of weeding (water vegetation) and mowing (bank) do you essentially perform in one operation?

The following schemes describe different types of water management. Please tick the scheme (box to the left) which is most likely to be performed by you. Again, feel free to check off two schemes and note which is most likely to be used for close-to-nature management, and which is most likely to be used for far-from-nature management. Examples:
Scheme below-left: The mowing of one bank takes place in one operation (alternate mowing) as well as the complete weeding of the bed.
Scheme central-center: The mowing of one bank takes place in one operation (alternate mowing) as well as the weeding of the bed, but here with the omission of refugial zones.

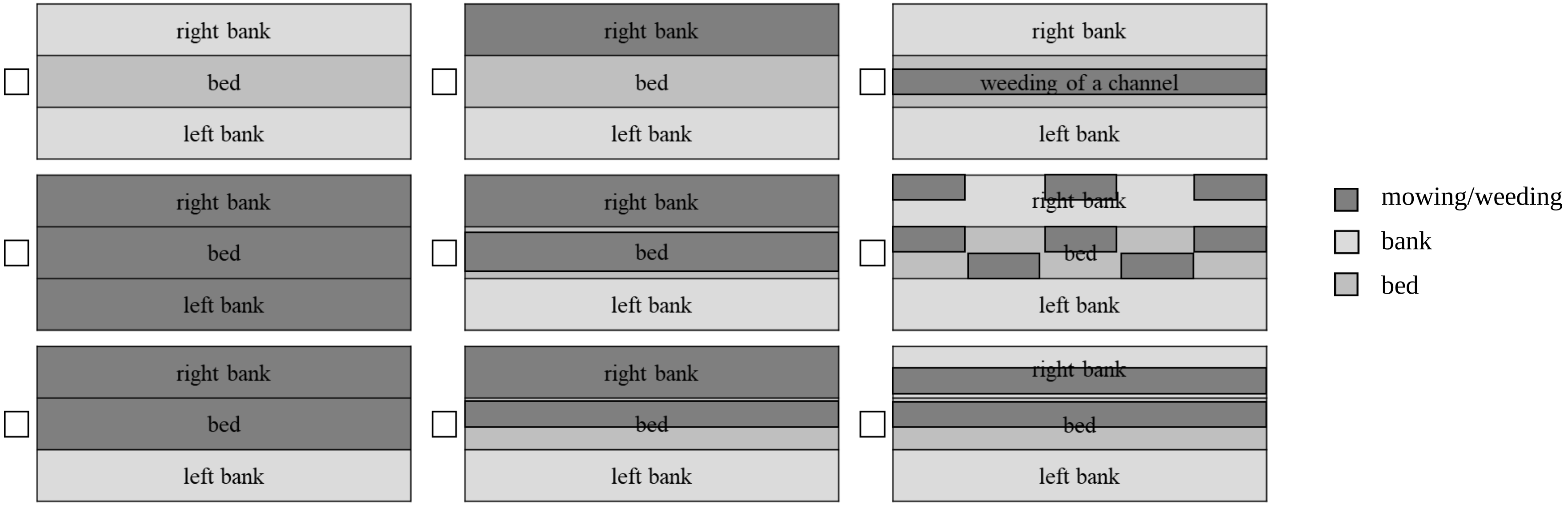

Question 12:

If performed: In which direction are weeding actions carried out?

☐ with the current

☐ against the current

☐ other: ____

Biological Background

Question 13:

Are any of the waterbodies you manage located in one or more of the following protected area(s)? - Your statements will be treated confidentially, as already mentioned on page 1.

☐ SAC (special area of conservation) according to the Habitats Directive

☐ nature conservation area

☐ other: ____

Question 14

If known: Do chemical/biological water analyses take place at specific waterbodies you manage?

☐ yes

☐ yes, chemical

☐ yes, biological

☐ no

Question 15:

Is the distribution of strongly/especially protected species clarified at managed waterbodies?

☐ notifications of the lower nature conservation authority

☐ commissioned ecological opinion

☐ other: ____

Question 16:
Are you familiar with the damselfly species *Coenagrion mercuriale*?

☐ yes
☐ yes, furthermore I know its habitat requirements
☐ no

Question 17:
Are nature conservation concerns considered in the application and selection of water management actions?

☐ yes
☐ yes, with regard to specific species: ____
☐ no, p.r.n. give reasons: ____

CONCERNING THE CLIENT

Question 18:
For which client are you working? (e.g. municipality)

____

Question 19:
Since when are you working for the named client?

☐ 0-2 years
☐ 3-5 years
☐ 6-10 years
☐ 11-15 years
☐ > 15 years

Question 20:
If known: Are there any larger *Coenagrion mercuriale* populations (>10 individuals seen) in the managed area?

☐ yes

☐ no

Question 21:
Would you be willing to review and, if necessary, implement a new water management concept for individual reaches with regard to the protection of the damselfly *Coenagrion mercuriale* and subsequently other species? An impact control will then be carried out by my side.

☐ Yes, review and implementation if necessary

☐ Yes, only review

☐ no

☐ other: ____

Question 22:
In case of any queries on my part, what is the best way to reach you (phone, email)?

____

Thank you very much for your time. You have been very helpful to me. If you have any questions or suggestions, please feel free to contact me.

Isabelle Immerschitt
